# Technology Acceptance and Usability of the BrainFx SCREEN in Canadian Military Members and Veterans With Posttraumatic Stress Disorder and Mild Traumatic Brain Injury: Mixed Methods UTAUT Study

**DOI:** 10.2196/26078

**Published:** 2021-05-13

**Authors:** Chelsea Jones, Antonio Miguel-Cruz, Suzette Brémault-Phillips

**Affiliations:** 1 Heroes in Mind, Advocacy and Research Consortium Faculty of Rehabilitation Medicine, University of Alberta Edmonton, AB Canada; 2 1 Field Ambulance Physical Rehabilitation Department Canadian Forces Health Services Department of National Defense Edmonton, AB Canada; 3 Leiden University Medical Centre Leiden Netherlands; 4 Department of Occupational Therapy Faculty of Rehabilitation, University of Alberta Edmonton, AB Canada; 5 Glenrose Rehabilitation Hospital Research Innovation and Technology Glenrose Rehabilitation Hospital Edmonton, AB Canada

**Keywords:** NCAT, PTSD, cognitive assessment, cognition, executive function, technology acceptance, UTAUT, Canadian Armed Forces, mTBI, concussion, digital health, neuropsychology, neurology, post concussive symptoms, military

## Abstract

**Background:**

Canadian Armed Forces service members (CAF-SMs) and veterans exhibit higher rates of injuries and illnesses, such as posttraumatic stress disorder (PTSD) and traumatic brain injury, which can cause and exacerbate cognitive dysfunction. Computerized neurocognitive assessment tools have demonstrated increased reliability and efficiency compared with traditional cognitive assessment tools. Without assessing the degree of technology acceptance and perceptions of usability to end users, it is difficult to determine whether a technology-based assessment will be used successfully in wider clinical practice. The Unified Theory of Acceptance and Use of Technology model is commonly used to address the technology acceptance and usability of applications in five domains.

**Objective:**

This study aims to determine the technology acceptance and usability of a neurocognitive assessment tool, which was titled BrainFx SCREEN, among CAF-SMs and veterans with PTSD by using the Unified Theory of Acceptance and Use of Technology model.

**Methods:**

This mixed methods embedded pilot study included CAF-SMs and veterans (N=21) aged 18-60 years with a diagnosis of PTSD who completed pre- and postquestionnaires on the same day the BrainFx SCREEN was used. A partial least squares structural equation model was used to analyze the questionnaire results. Qualitative data were assessed using thematic analysis.

**Results:**

Facilitating conditions, which were the most notable predictors of behavioral intention, increased after using the BrainFx SCREEN, whereas effort expectancy decreased. Performance expectancy, effort expectancy, and social interaction were not factors that could predict behavioral intention. Participants who reported a previous mild traumatic brain injury were significantly more likely to report current symptoms of cognitive impairment. The BrainFx SCREEN is a feasible, usable, and accepted assessment tool for CAF-SMs and veterans who experience PTSD.

**Conclusions:**

As military health care systems integrate technological innovations to improve the services and care provided, research must continue to address the acceptability and use of these novel assessments and interventions.

## Introduction

### Background

Canadian Armed Forces service members (CAF-SMs) and veterans exhibit higher rates of injuries and illnesses, such as posttraumatic stress disorder (PTSD), depression, anxiety, sleep disorders, and mild traumatic brain injury (mTBI), which can cause and exacerbate cognitive dysfunction [1,2]. Numerous studies conducted in Canada, the United States, and the United Kingdom demonstrate a high prevalence of mTBI and PTSD as comorbidities specific to deployments during the War on Terror (2001-2013) [3-5]. The co-occurrence of traumatic brain injuries (TBIs) and PTSD can arise from the same or separate traumatic incidents [3].

When mTBI symptoms persist for longer than 3 months, they may be referred to as postconcussive symptoms (PCSs) [6]. In a study assessing CAF-SMs with mTBI from deployments in Iraq and Afghanistan during the War on Terror, PCS was present in 21% of those with less severe forms of mTBI and in 27% of those with more severe forms of mTBI [7]. The rates of PTSD among Canadian veterans have been estimated to be 16% [8]. Interestingly, after adjustment for confounding variables, mTBI was found to have no significant association with PCS relative to non-TBI injury [7]. Mental health conditions, such as combat-related PTSD, had a strong association with reporting three or more PCSs [5,7]. Identifying if symptoms are related to mTBI and/or a concurrent mental health diagnosis is difficult, as many of the symptoms attributed to these conditions overlap. Symptoms often described as PCS in patients with mTBI may be better explained from a psychological standpoint and may be more likely to be caused by PTSD [9]. Cognitive dysfunction is a common symptom experienced by many CAF-SMs and veterans who have experienced PTSD, mTBI, and/or a host of other comorbid conditions.

### Cognitive Dysfunction and Assessment

Cognition is a broad construct that refers to information processing functions carried out by the brain [10]. Such functions include attention, memory, executive functions, comprehension, speech [11], calculation ability [12], visual perception [13], and praxis skills [14,15]. Cognition is instrumental in human development and the ability to learn, retain, and use new information in response to everyday life and is integral to effective performance across a broad range of daily occupations, such as work, educational pursuits, home management, self-regulation, health management, and leisure activities [15]. Reduced cognitive functioning can detrimentally affect a person’s relationships and cause mental and emotional distress [15,16]. Within the military context, cognitive dysfunction can potentially result in decreased efficiency and effectiveness and increased risk of harm to self, the unit, and the mission [2].

Owing to the cognitive challenges and dysregulation that can be caused by PTSD, cognitive assessment and screening is important to enable clinicians to recommend treatment, referrals, and advise on a CAF-SM’s or veteran’s safety in activities of daily living, which may include military activities [16,17]. Reliable, valid, specific, and function-based cognitive screening and assessment practices are essential for determining the effective interventions to improve cognitive functioning [17]. Computerized neurocognitive assessment tools (NCATs) are widely used in other global militaries and have multiple benefits, including increased inter- and intrarater reliability, ease of administration, reduced time to administer, and ease of calculation and analysis of results [18]. One such tool that is being trialed within the Canadian Forces Health Services (CFHS) is the BrainFx SCREEN.

### BrainFx SCREEN

The BrainFx SCREEN is a function-focused, Canadian-made screen that addresses neurofunction through a digital interface on a tablet [19]. On the basis of its more comprehensive predecessor, BrainFx 360, BrainFx SCREEN has a 10- to 15-minute duration and is administered by a health care professional trained as a Certified BrainFx Administrator (CBA) via a touch tablet to set a baseline or to determine if a further assessment or test is needed [19]. The BrainFx SCREEN has 15 tasks within seven domains of cognition, which include (1) overall skill performance, (2) sensory and physical skill performance, (3) social and behavioral skills performance, (4) foundational skills performance, (5) intermediate skills performance, (6) complex skills performance, and (7) universal skills [19]. These seven domains encompass a variety of cognitive skills, including different areas of memory, attention, visuospatial, and executive functions [19]. The BrainFx SCREEN is a new and innovative tool based on the BrainFx 360 assessment; as such, it has not been researched for validity and reliability as its predecessor has. The BrainFx SCREEN also collects a variety of demographic and health information, including level of education, presence of other comorbidities including mTBI, chronic pain, and other mental health diagnoses, current level of fatigue, presence of sleep difficulties, and presence of self-perceived neurofunctional deficits. The BrainFx 360 assessment has been subjected to reliability and validity testing, and current evidence demonstrates that this comprehensive assessment has promising validity, reliability, and sensitivity, with a focus on neurofunction [20] (Sergio L, unpublished data, 2014). The BrainFx SCREEN has undergone widespread uptake within Canada and the United States but has yet to be tested based on evidence-based models or frameworks for technology acceptance.

### Technology Acceptance and Usability in Health Care and Military Contexts

Technology offers health care professionals a variety of benefits from improving effectiveness, efficiency, and potential engagement in record keeping, assessments, and interventions. As such, the acceptance of such technologies by health care professionals, and their patients, is an important topic of interest for both practitioners and researchers [21]. Without technology acceptance and acceptable usability for the user, technological assessments and interventions may not be adopted in clinical practice despite its effectiveness. The evaluation of acceptance and usability of emerging technology is integral to advance best practices in health care [22].

Owing to some of the fundamental differences in military culture, environment, and contexts, the relationship between users and technologies, and the variables influencing this, may need to be considered separately from civilian relationships with technology. Many military organizations’ approach to technology is to measure and maximize operator performance to increase system efficiency, which translates to success in military missions [23]. It is unknown if current models and frameworks of technology acceptance and usability are applicable to military populations, as the relationship between military personnel and organizations is not consumer based. It may also be presumed that the performance and functionality of technology are prioritized over comfort and esthetics [23].

Regardless of the potential differences in the relationship between the user and technology in a military context, the use of eHealth and mobile health (mHealth) innovations is becoming widespread within military and veteran populations [24,25]. This has been amplified by the recent COVID-19 pandemic when virtual health solutions have become increasingly common in all health care practices, including those in military environments. Although most studies addressing technology attitudes, beliefs, acceptance, and usability within military and veteran populations are US based, current evidence suggests that the military population is willing to use digital health and mHealth technologies [25-27]. Regardless of the context for technological innovation, adequate technology acceptance and usability is key to its uptake within that environment and culture. Before addressing the facilitators and barriers to the usability of a technological innovation, it is helpful to directly or indirectly assess technology acceptance within different user groups within their context using a framework or model.

### The Unified Theory of Acceptance and Use of Technology Model

**Figure 1:**
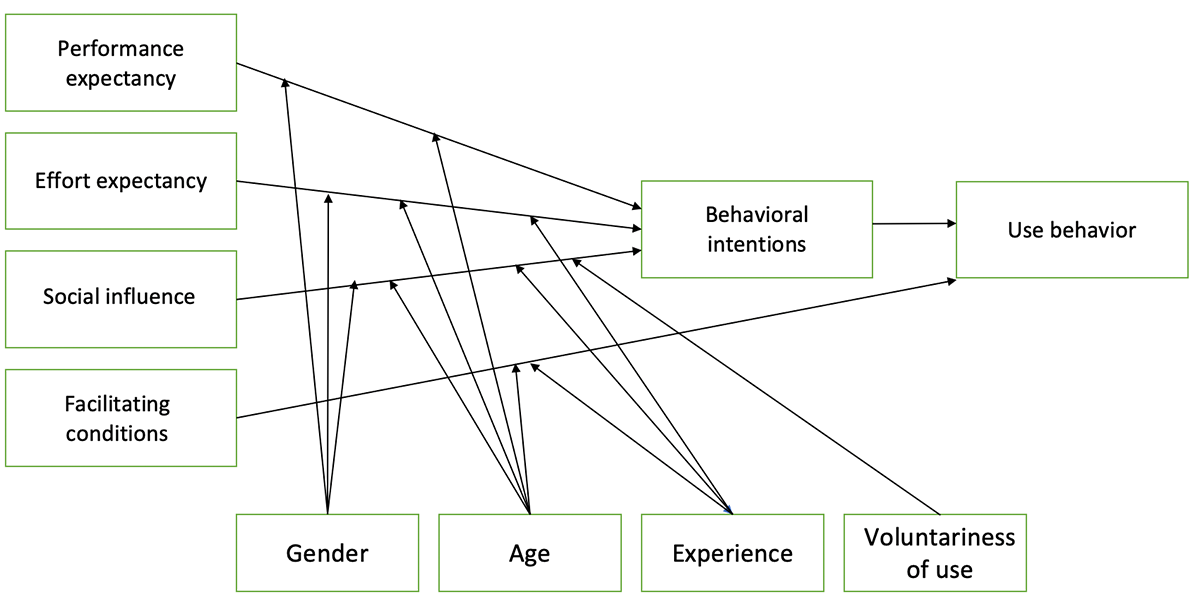
The Unified Theory of Acceptance and Use of Technology model.

The Unified Theory of Acceptance and Use of Technology (UTAUT) model was developed based on previous theories and models for acceptance and adoption of technologies and consumer products that address the perceived technology acceptance of a user group with the goal of predicting usage behavior (Figure 1) [28]. The UTAUT has been demonstrated to explain as much as 70% of the variance in intention to use technology compared with its technology acceptance model predecessors [28].

This model was developed from the point of view of the implementation of new technologies in practice within organizations on individuals rather than technology for mass consumer consumption [29,30]. The UTAUT model addresses the perceived expectations of technological acceptance of new technology in five constructs: performance expectancy (PE), effort expectancy (EE), social influence (SI; direct determinants of behavioral intention [BI]), facilitating conditions (FC), and BI, which is the direct impact on use behavior [28]. The UTAUT is a model that is commonly tested using partial least square (PLS) structural equation modeling (SEM) and is an example of a reflexive PLS path model [28]. The exogenous latent variables (PE, EE, and SI) affect the endogenous latent variable (BI), which affects the construct of use [28]. In addition, FC can also have a direct effect on use [28]. Moderator variables, which include age, gender, experience, and voluntariness of use, also affect the interaction between the indicators and constructs [28,30].

BI is defined as the intention to use technology, and use is defined as the actual use [28]. BI predicts whether the technology in question will be adopted by the user in reality. The three direct determinants of BI to use technologies are PE, EE, and SI. PE is defined as the degree to which an individual believes that using the system will help the person attain gains in task performance [28]. The EE construct was defined as the degree of ease associated with the use of the system, and SI is the degree to which an individual perceives that important others believe they should use the new system [28]. FC have been defined as the degree to which an individual believes that an organizational and technical infrastructure exists to support the use of the system [28]. FC, PE, and EE are considered beliefs, or the information the person has about an object, and SI is considered the subjective norm [28]. The UTAUT has a well-established construct and content validity. Validity is more likely to be influenced by bias and other factors, including those unique to research with military populations.

The UTAUT model is most commonly used in civilian populations. As military contexts necessitate unique and varying relationships between user groups and technology, it is unknown whether the UTAUT model could be an accurate representation of technology acceptance and usability among military members and other secondary or tertiary users. The perspective of the end user and primary user, the military member, is not always measured or even considered because global effectiveness is prioritized over individual preferences [23]. Within the military context, there is an intent that technological innovations can be used effectively, efficiently, safely, and confidently in support of the mission. Military personnel are expected to use technological innovations as directed. The personal preferences of military personnel are generally not as critical as they would be in commercial industries, unless safety is compromised. As the UTAUT was originally developed for an individualistic approach to measure technology acceptance and usability, it may not be applicable to military contexts [23,28]. The literature using the UTAUT model among military populations is scarce, and the model has not been used in the CAF context. The results of existing studies using the UTAUT among military populations demonstrate varying results, making it challenging to form a hypothesis for future studies.

The UTAUT has been used in more recent years as a model and framework for addressing technology use and acceptance in health care [22]. To date, most research in health technology using the UTAUT has involved the exploration of computerized medical records, where the primary intended user is the health care professional [31]. Studies that focus on the patient as the primary intended user are beginning to emerge in the literature with specific demographics, such as older adults, youth, and cardiac populations. These studies have evaluated the technology acceptance and usability of a multitude of digital and mHealth technologies, including health apps, wearable measurement technology, and virtual access to medical records. Hypotheses regarding the effect of the latent variables on BI and use have been formed regarding health care professionals as the primary intended users. Studies focusing on the patient as the primary intended user have demonstrated variable results, making the formation of a directional hypothesis challenging.

Despite the paucity of evidence-based information regarding technology acceptance models in military contexts, the UTAUT was chosen for use in this study because of its higher potential to explain variance and the fact that it has been used in health care studies. The technology acceptance and usability of NCATs from the perspective of the patient within a health care setting warrants evaluation, as questions of feasibility must be addressed before in-context clinical investigations regarding specificity, reliability, validity, and sensitivity can take place. Without addressing acceptance and usability, technological innovations may not be adopted or sustained. Although technology acceptance and usability testing are emerging in health care settings, the combination of a military context and its effects at multiple user levels warrants further exploration. The adoption of the BrainFx SCREEN within CFHS provides an opportunity to investigate technology acceptance and usability at the primary user level of the patient.

### Objective

This mixed methods pilot study aims to determine the technology acceptance and usability of a computer-based cognitive BrainFx SCREEN by CAF-SMs and veterans with combat-related posttraumatic stress disorder (crPTSD) using the UTAUT model. This study acknowledges CAF-SMs and veterans with crPTSD and/or mTBI as the primary intended users. Potential rejection of the BrainFx SCREEN by the CAF-SMs would provide important information and direction to CFHS on the way forward in addressing cognitive assessment with the BrainFx SCREEN as a tool. It was hypothesized that PE and FC would be the most influential variables for BI and use, respectively. It is also hypothesized that SI would have the least influence on BI.

### Research Model

**Figure 2:**
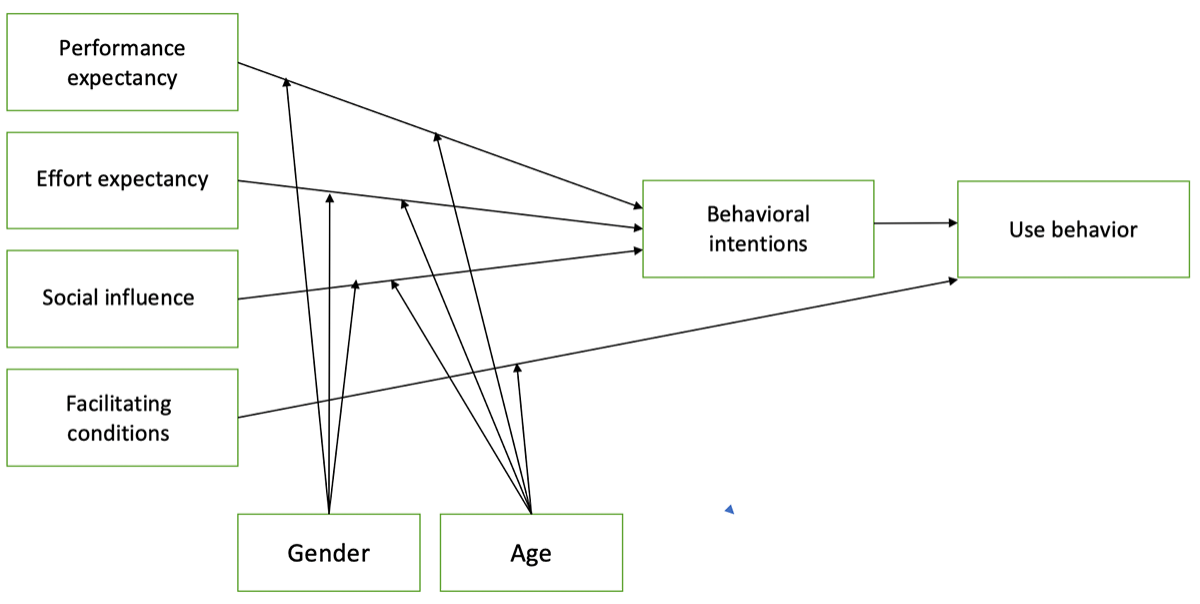
The Unified Theory of Acceptance and Use of Technology model with age and gender as the moderator variables.

Figure 2 shows the research model used in this study. The moderator variables of *experience* were removed because the BrainFx SCREEN is not meant to be used continuously or practiced with the goal of improving performance when used as an assessment tool. As the user is asked to complete the assessment by their clinician and is not a tool designed for regular use, the moderator of *voluntariness of use* was removed for the research model. Age and gender are the two moderator variables that remained in the original research model used in this study.

## Methods

### Study Design

This study of the technology acceptance of the BrainFx SCREEN was a mixed methods embedded study design with a quantitative prequasi-experimental or postquasi-experimental approach as the primary method of data collection and a qualitative thematic analysis secondary to this. This study was embedded in a larger clinical trial, which undertook a mixed methods, staggered entry randomized controlled trial (RCT) [32].

### Sample Size

The target sample size was set at a minimum of 32 CAF-SMs and/or veterans with crPTSD who would participate in the study to account for a 10% dropout rate, which would still allow for power at 24 participants. With four latent variables, for 80% significance at a 5% significance level, the sample size required for this study was 18 (R^2^=0.50 [33]).

### Recruitment and Sampling

Recruitment of regular and reserve CAF-SMs and veterans was conducted by word of mouth among potential participants and mental health service providers as convenience and snowball sampling. Service providers supporting CAF-SMs and veterans, after being informed of the study via word of mouth and institutional email, informed the patients who met the study inclusion and exclusion criteria. Potential participants who showed interest in participation were provided with a *Permission to Share Contact Information with the Research Team* form by their service provider. The completed forms were forwarded to the research team. The researchers then contacted the potential participants via phone or email with a request for them to meet with the research team to learn more about the study and be evaluated to confirm eligibility to participate. Voluntary verbal and written informed consents were obtained from all CAF-SMs and veterans participating in the study. In addition, the BrainFx SCREEN has an additional digital informed consent form that is required before partaking in the screen.

### Inclusion and Exclusion Criteria

Participants included regular and reserve CAF-SMs and veterans aged 18-60 years under the care of a mental health clinician or service provider working at or associated with Canadian Forces Base Edmonton, an Operational Stress Injury Clinic in Edmonton and Calgary, Alberta, or Veterans Affairs Canada. All participants met the Diagnostic and Statistical Manual of Mental Disorders, 5th Edition (DSM-5) [34] criteria for PTSD diagnosis and had a score of ≥30 or higher on the Clinician-Administered PTSD Scale for DSM-5 Worst Month version. Participants were classified as having treatment-resistant crPTSD, which indicated that they had previously not responded to at least two types of evidence-based treatments, of which at least one must have been a psychotherapeutic intervention. Participants were stable on their current psychotropic medication for at least 4 weeks before entering the study. Individuals with comorbidity (ie, mTBI) were included if they satisfied the other inclusion or exclusion criteria. Participants were English speaking and were able to provide informed written consent.

### Measurements and Instruments

In total, two UTAUT questionnaires specific to the patient population were developed specifically for this study. Version 1 (T0) includes questions in the future tense, whereas version 2 (T1) includes the same questions but is modified to reflect the past tense. The 12-question outcome measures are based on a Likert scale with a score of 1-7 assigned to each question, with 1 being *strongly disagree* and 7 being *strongly agree*. A Likert scale with 7 points was used, as the original UTAUT questionnaire by Venkatesh et al [28] used a 7-point scale. The maximum score was 84, and the minimum score was 12. The 12 included questions addressed the five different constructs of the UTAUT (2 PE, 3 EE, 3 SI, 3 FC, and 1 BI) that influence the use of a technological innovation. Gender and age demographic information was also collected via the UTAUT questionnaire, as they are modifier variables within the UTAUT model.

Two additional open-ended questions were asked as part of both questionnaires: (1) What did you like most about the BrainFx SCREEN? (2) What did you like the least about the BrainFx SCREEN?

### Data Collection

The BrainFx SCREEN and both UTAUT questionnaires were completed on the same day within 30 minutes. The BrainFx SCREEN and UTAUT questionnaires were administered by the CBA. First, the participants were provided with an explanation of the purpose of the BrainFx SCREEN by the CBA. Second, the participants were presented with the BrainFx SCREEN tablet and asked to read the introduction screen and acknowledge that they understood the purpose of the assessment. They were then presented with a paper version of the first UTAUT questionnaire (version 1; future tense, intended to measure expectations of the technology). After completing this questionnaire, the full BrainFx SCREEN was executed on the tablet. On completion of this, the second paper-based UTAUT questionnaire (version 2; past tense, intended to measure actual intention to use technology) was completed by the participant.

### Data Analysis

PLS-SEM was used for this study based on the UTAUT, which uses a reflexive path model. The expectations from T0 and the actual experience from T1 were statistically analyzed using PLS-SEM with both a within-sample path model and a pre or post analysis (multigroup analysis [MGA]).

SEM is considered a second-generation technique of multivariate analysis that allows researchers to incorporate unobservable variables measured indirectly by indicator variables [35]. PLS-SEM is variance based, as it accounts for the total variance and uses this to estimate parameters [35]. In this method of analysis, the algorithm computes partial regression relationships in the measurement and structural models using ordinary least squares regression [35,36]. In an exploratory study such as this, data analysis is concerned with testing a theoretical framework from a prediction perspective, making PLS-SEM an ideal method for analysis [36].

The path model must be analyzed through measurements and structural model assessments [35,36]. Reflexive measurement models were evaluated based on internal consistency (Cronbach α), convergent validity (average variance extracted [AVE]), and discriminant validity (cross-loading analysis, Fornell-Larcker criterion analysis, and Heterotrait-Monotrait ratio [HTMT]) [35]. Evaluation of the structural model included an analysis of collinearity, significance, the coefficients of determination (R^2^), size and significance of the path coefficients, effect size (*f*^2^), and predictive relevance (*q^2^*). Goodness-of-fit was not assessed, as this is an exploratory PLS path model with both reflexive (measurement model) and formative (structural model) components, rendering current model fit measurements unnecessary and inappropriate [35].

As PLS-SEM does not assume that data are normally distributed, it relies on a nonparametric bootstrap procedure to test the significance of estimated path coefficients in PLS-SEM. With bootstrapping, subsamples are created with randomly drawn observations from the original set of data (with replacement) and then used to estimate the PLS path model [37]. In this study, only participant data that were complete with pre- and postresults were included; therefore, a strategy to manage missing data was not required.

SmartPLS [38] was used for the PLS analysis. The maximum iterations were set at 300 with +1 for the initial value for all outer loadings and the path weighting scheme and the stop criterion at 1×10^7^. A minimal number of bootstrap repetitions needed depends on the desired level of accuracy, the confidence level, the distribution of the data, and the type of bootstrap CI constructed [39]. It is commonly accepted that 5000 bootstrap repetitions meet this minimum threshold [40]. Basic bias-corrected bootstrapping was performed with 5000 samples at a significance level of *P*<.05. SPSS (2017; IBM Corporation) [41] was used to analyze descriptive statistics (mean and SD), frequency counts, Pearson Chi-square test, and the Harman single-factor test [42,43]. Webpower [44] was used to verify the nonnormality of the data before analysis. Qualitative data from the questionnaires were assessed using NVivo (QSR International) [45] software to identify key themes. A concurrent parallel approach following a data transformation model was used in the data analysis process to converge the data to compare and contrast quantitative statistical results with qualitative findings [46].

## Results

### Overview

Demographic information of the sample (N=21) is presented in Table 1. The sample was largely male (n=20), which prevented the use of gender as a moderator variable in the research model. In addition, the age of the participant (young or middle aged) did not demonstrate to have an effect in the research model and was therefore removed for the final PLS model. The psychometric properties of the raw data of the survey items used to measure the latent variables are presented in Tables 2 and 3. The difference between the means of the pre- and postscores was a 2.6% increase (Table 3). When pre- or postscores indicate a less than 5% difference in change, this is indicative that the expectations of the participants regarding technological innovation were met within the constructs tested [28].

**Table 1:** Sample demographic information (N=21).

Characteristics			Participant, n (%)
**Gender**			
	Male	20 (95)	
	Female	1 (5)	
**Age (years)**			
	18-34 (young)	10 (48)	
	35-60 (middle age)	11 (52)	
**Military employment status**			
	Regular force member	8 (38)	
	Veteran	13 (62)	
**Education**			
	High school diploma	21 (100)	
	Diploma	6 (29)	
	Degree	1 (5)	
	Graduate degree	1 (5)	
	Missing	4 (19)	
Previous mild traumatic brain injury or traumatic brain injury			14 (67)
Current cognitive dysfunction			18 (86)

**Table 2:** Psychometric values of indicator variables.

Exogenous latent variables (indicators)			Value, mean^a^ (SD)		Value, median^b^
**Performance expectancy (two indicators)**					
	1. Using the BrainFx SCREEN would improve my medical condition.	4.143 (1.424)		4	
	2. Using the BrainFx SCREEN would have a positive effect on my medical condition.	4.524 (1.292)		4	
**Effort expectancy (three indicators)**					
	1. I believe my interaction with the BrainFx SCREEN will be clear and understandable.	5.5 (1.383)		6	
	2. Interaction with the BrainFx SCREEN will be easy for me.	5.452 (1.301)		5	
	3. I believe that it is easy to get the BrainFx SCREEN to do what I want it to do.	5.119 (1.382)		6	
**Social influence (three indicators)**					
	1. I would use the BrainFx SCREEN because my colleagues will use it too, to improve their medical condition.	4.5 (1.502)		4	
	2. People who are important to me think that I should be involved in using the BrainFx SCREEN.	4.667 (1.14)		4	
	3. In general, my organization has supported my involvement in utilizing the BrainFx SCREEN.	4.833 (1.057)		4	
**Facilitating conditions** **(three indicators)**					
	1. I believe specialized instruction concerning the interaction with the BrainFx SCREEN will be available to me.	5.81 (1.063)		6	
	2. I believe guidance will be available to me during my utilization of the BrainFx SCREEN.	6.119 (1.234)		6	
	3. I have the necessary resources to use the BrainFx SCREEN.	5.881 (1.108)		6.5	
**Behavioral intention (one indicator)^c^**					
	1. I am willing to use the BrainFx SCREEN in the future.	6.333 (0.845)		7	

^a^Raw mean scores of items within scale where each item is measured on a 7-point Likert scale (1=strongly disagree; 7=strongly agree). The higher the indicator score, the more agreement with the statement.

^b^Median scores of each question.

^c^Single indicator.

**Table 3:** Descriptive analysis of total pre- or postscores.

Total score	Value, mean (SD)^a^	Value, median^b^ (range)
Pre (T0)	62.05 (8.87)	60 (48-76)
Post (T1)	63.71 (9.71)	64 (42-84)

^a^Mean total and SD of pre and post raw scores.

^b^Median of the means of pre and post raw scores.

In addition, a Pearson Chi-square test was used to measure whether participants who reported experiencing an mTBI were more likely to report ongoing cognitive symptoms. Participants who reported a previous mTBI were significantly more likely to report currently experiencing symptoms of cognitive impairment (*P*<.001).

### Measurement Model

The results of the measurement model evaluation, including the factor analysis, internal consistency (Cronbach α), convergent validity (AVE), and composite reliability, are presented in Table 4. The factor indicators, which are known as the outer loadings or reflexive indicator loadings, should be ≥0.5 to demonstrate that the indicator variable is a good measurement of the latent variable [47]. Only one outer loading for SI was below this threshold, indicating good indicator reliability (Table 4). All the latent variables, with the exception of SI, demonstrated values of above 0.70 for both Cronbach α and AVE, which indicated the good validity and reliability of the latent variables [35]. A single-item construct, such as BI, is not represented by a multi-item measurement model; thus, the relationship between the single indicator and latent variable is 1 [35]. As there are no established criterion variables to correlate with the BI indicator, criterion validity and reliability cannot be determined for this construct [35]. Composite reliability is presented in Table 4, and all values, with the exception of SI, were ≥0.7, which is acceptable.

**Table 4:** Measurement model.

Latent and indicator variables		Outer loadings^a^	Cronbach α^b^	Average variance extracted^c^	Composite reliability^d^
**BI^e,f^**			1.000	1.000	1.000
	1. BI indicator	1.000			
**EE^g^**			.857	0.776	0.912
	1. EE indicator	0.866			
	2. EE indicator	0.926			
	3. EE indicator	0.849			
**FC^h^**			.874	0.798	0.922
	1. FC indicator	0.885			
	2. FC indicator	0.928			
	3. FC indicator	0.866			
**PE^i^**			.885	0.875	0.933
	1. PE indicator	0.881			
	2. PE indicator	0.987			
**SI^j^**			.446	0.402	0.559
	1. SI indicator	−0.011			
	2. SI indicator	0.601			
	3. SI indicator	0.919			

^a^Outer loadings of ≥0.5 indicate indicator reliability.

^b^With a reflective model, internal consistency is measured by Cronbach α; values of ≥.7 indicates good indicator reliability.

^c^Average variance extracted values of ≥0.5 indicates convergent validity.

^d^Composite reliability values of ≥0.5 indicates good internal consistency.

^e^BI: behavioral intention.

^f^Single indicator.

^g^EE: effort expectancy.

^h^FC: facilitating conditions.

^i^PE: performance expectancy.

^j^SI: social influence.

To evaluate discriminant validity, cross-loading, the Fornell-Larcker criterion, and HTMT (Table 5) were used. These measures demonstrated good discriminant reliability for all latent variables, except for SI. FC demonstrated the highest correlation with BI based on this analysis. Potential common method bias was assessed with the Harman single-factor test, yielding cumulative and variance loadings under 50% (34.43%).

**Table 5:** Discriminant validity.

Measure		Latent variables^a^				
		BI^b,c^	EE^d^	FC^e^	PE^f^	SI^g^
**Fornell-Larcker criterion**						
	BI^b^	1.000	—^h^	—	—	—
	EE	0.467	0.881	—	—	—
	FC	0.736	0.564	0.893	—	—
	PE	0.052	0.343	0.025	0.935	—
	SI	0.340	0.173	0.393	0.325	0.634
**Heterotrait-Monotrait ratio**						
	BI^b^	—	—	—	—	—
	EE	0.495	—	—	—	—
	FC	0.776	0.654	—	—	—
	PE	0.045	0.339	0.122	—	—
	SI	0.336	0.403	0.438	0.985	—

^a^Diagonals are the square root of the average variance extracted of the latent variables and indicate the highest in any column or row.

^b^Single indicator.

^c^BI: behavioral intention.

^d^EE: effort expectancy.

^e^FC: facilitating conditions.

^f^PE: performance expectancy.

^g^SI: social influence.

^h^Not applicable.

**Figure 3:**
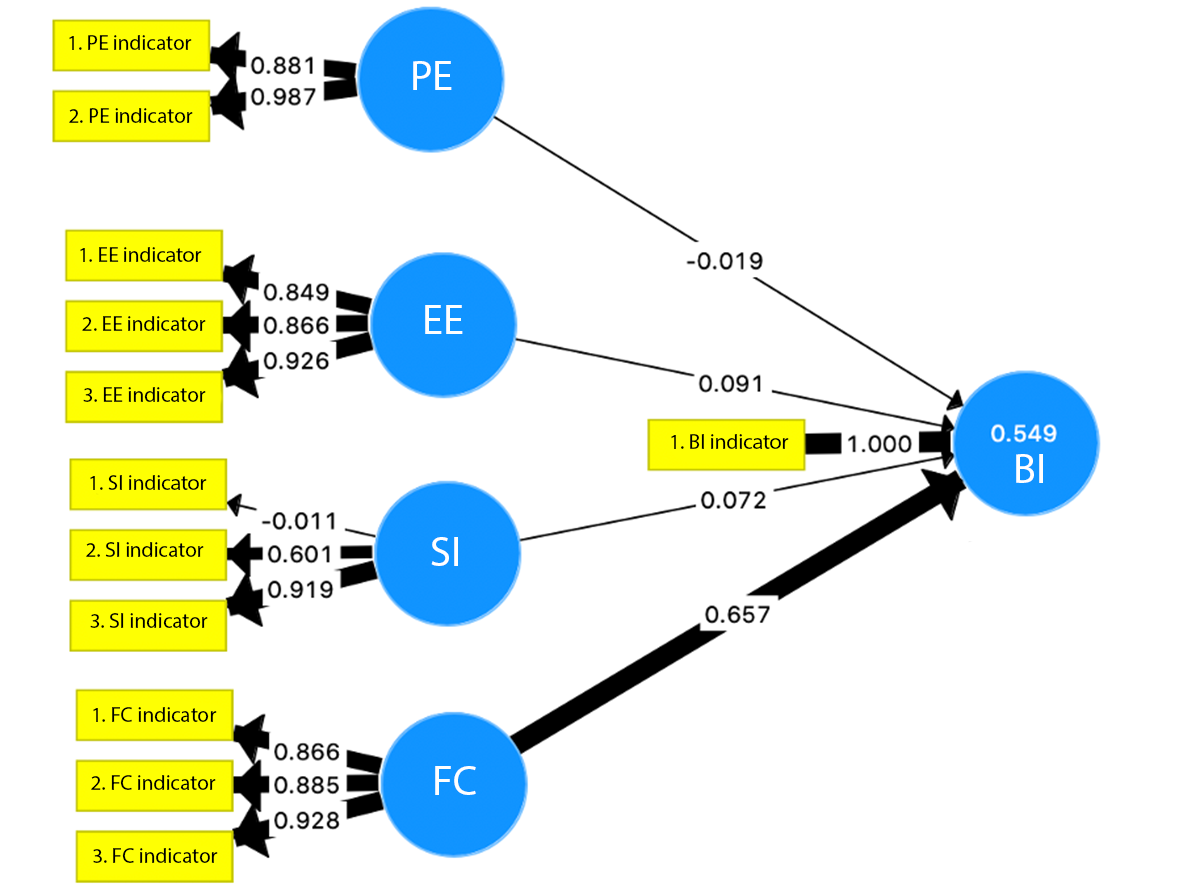
Path analysis model of the Unified Theory of Acceptance and Use of Technology for predicting BI. Facilitating conditions is the largest predictor of BI (path coefficient=0.657; R2=0.549). The thicker the arrow, the larger the effect on the variable or construct in the measurement or structural model. BI: behavioral intention; EE: effort expectancy; FC: facilitating conditions; PE: performance expectancy; SI: social influence.

The measure of lateral collinearity of the structural model demonstrated inner variance inflation factor values below 5 for all latent variables. The coefficient of determination (R^2^) measures the proportion of variance in a latent endogenous variable that is explained by other exogenous variables expressed as a percentage. The explained variance (R^2^) of the structural model was 0.549, indicating that >50% of BI was explained by this model and moderate predictive accuracy. The effect size (*f*^2^) for each latent variable is listed in Table 3. On the basis of this analysis of the structural model, the largest path coefficient and effect size were for FC, indicating that it was the strongest predictor of BI (Table 6 and Figure 3).

**Table 6:** Structural model evaluation and hypothesis testing.

Relationship^a^	Standard β	SE	Critical *t* value	Effect size, *f*^2^	Predictive relevance, *q*^2^	95% CI
Performance expectancy - >BI^b^	.013	0.11	0.176	0.001^d^	−0.04	−0.215 to 0.212
Effort expectancy - >BI	.108	0.153	0.598	0.01	0	−0.179 to 0.409
Social influence - >BI	.075	0.108	0.669	0.008	−0.03	−0.152 to 0.277
Facilitating conditions - >BI	.643	0.166	3.950^c^	0.492	0.443	0.285 to 0.95

^a^Effect size (*f*^2^) and predictive relevance (*q*^2^) values under 0.02 denote small effect size or predictive relevance, whereas values of >0.35 indicate large effect size or predictive relevance [33].

^b^BI: behavioral intention.

^c^*P≥*.05.

On the basis of the MGA, there was a statistically significant increase (*P*=.007) in the scores for FC in the version 2 UTAUT questionnaire (post: T1) data compared with the version 1 UTAUT questionnaire (pre: T0) data. A statistically significant decrease in EE was noted in the version 2 UTAUT questionnaire (post: T1) data compared with the version 1 UTAUT questionnaire (pre: T0) data, where the latent variable EE was a significant predictor of BI within the pregroup but not the postgroup (Table 7; *P*=.03). Combined, this rendered EE to not be statistically significant in predicting BI. There were no statistically significant changes in the PE or SI pre- or postgroups (Table 7).

**Table 7:** Pre- or postmultigroup analysis.

Latent variable	Critical *t* value	*P* value
Performance expectancy	0.008	.99
Effort expectancy	2.355	.03^a^
Social influence	0.173	.86
Facilitating conditions	2.997	.007^a^

^a^Significant at *P*≤.05.

Finally, a brief thematic analysis was conducted by analyzing the responses to the open-ended questions from the UTAUT questionnaires (pre and post). The first two themes, likes and dislikes, were imposed on the data, whereas the third theme, the unclear purpose of cognitive assessments, arose inductively. The qualitative results were triangulated with the quantitative data and discussed further (Table 8).

**Table 8:** Thematic analysis results of qualitative questions from the Unified Theory of Acceptance and Use of Technology questionnaire.

Categories			Participant statements
**Likes**			
	Challenges the brain	“Challenged myself to multitask, test my short-term memory.”	
	Fun, engaging, and interactive	“Interaction with tablet. No writing. Fun.”	
	Easy to use	“Ease of use.”	
	Quick to complete	“Quick.”	
	Clear instructions	“Clear Instructions.”	
**Dislikes**			
	Math questions not enjoyable	“I hate math.”	
	Fear of the unknown	“(I have) anxiety about what it will be like.”	
	Screen sensitivity	“Touch screen delay, would rather use paper.”	
	Clarity of instructions	“Instructions not clear.”	
	Difficult to predict what stimuli can be a trigger	“Disturbing images”	
Unclear purpose of cognitive assessments			“Alternative treatment, mood alteration.”; “Help[ed] me to get rid of my anger.”

## Discussion

### Principal Findings

The UTAUT model was used as the theoretical foundation for understanding the BI of CAF-SMs and veterans with crPTSD to use the BrainFx SCREEN. FC were the most notable predictor of BI and increased after using the BrainFx SCREEN, whereas EE decreased. PE, EE, and social interaction were not factors predicting BI. On the basis of the study results, the BrainFx SCREEN appears to be a feasible, usable, and accepted assessment tool for CAF-SMs and veterans who experience PTSD.

A number of notable findings from this mixed methods pilot study warrant consideration. Demographically, 67% (14/21) of participants reported a previous mTBI or TBI as comorbid with their PTSD, and those who reported a previous mTBI or TBI were significantly more likely to report currently experiencing symptoms of cognitive impairment. The relationship between PTSD and mTBI, as well as its effect on cognition, is complex and continues to be a topic of research that is being explored among military and veteran populations. The most recent literature points to symptoms of PCS being largely attributed to PTSD as opposed to mTBI pathologies. If PCS are mostly attributable to mental health conditions in those with co-occurring mTBI, it would be assumed that those with and without past mTBI or TBI would report subjective cognitive impairment at the same rate.

Overall, CAF-SMs and veterans rated all the latent variables (PE, EE, FC, and SI) and BI favorably for the BrainFx SCREEN. The lowest mean latent variable score was for PE (4.334), whereas the highest was for BI (6.333), indicating that the participants generally agreed or strongly agreed with the statements made in the UTAUT questionnaires. The results of the PLS-SEM analysis demonstrated good internal consistency, convergent validity, composite reliability, and discriminant validity of the indicators, except for SI. The model explained 50% of BI, which indicated moderate predictive accuracy; however, the analysis of the structural model indicated that only FC had a significant effect on BI. FC had the largest path coefficient and effect size, indicating that it was the strongest predictor of BI. A statistically significant increase in FC and a decrease in EE were noted in the pre- and post-MGA. The less than 5% (2.6%) change in the pre- and postscores indicated that the expectations of the BrainFx SCREEN were generally met. The pre- and postchanges in the other latent variables were not significant. 

The analysis of the open-ended questions revealed a number of themes that could be attributed to the latent variables of the UTAUT and BI as a construct. To understand the results of the PLS-SEM and qualitative data, triangulation can provide a clearer explanation of why the relationships in the path model exist [46].

As previously mentioned, PE refers to the degree to which an individual believes that using the system will help the person attain gains in performance [28]. In the context of the BrainFx SCREEN, cognitive functioning in different neurofunctional domains is measured [19]. It is integral to the validity of the BrainFx SCREEN that the participant does not receive any feedback on their performance from either the CBA or the software and platform. The participants were limited to their intrinsic subjective insight to speculate their performance, which may be a logical explanation as to what PE did not register as an important factor in BI and did not demonstrate a significant pre- or postchange.

SI is the degree to which an individual perceives that important others believe that they should use the new system [28]. As the BrainFx SCREEN was performed within a research study with only a CBA present and confidentiality maintained, it is unlikely that the participants perceived SI specifically to the technology. This was demonstrated to be an accurate hypothesis, as SI was the least influential latent variable in the prediction of BI. 

EE is the degree of ease associated with the use of a system [28]. Many of the *likes* of the participants fell into the category of EE, including that the BrainFx SCREEN was *quick* and *easy to do*. Comments obtained from participants written in answer to open-ended questions in the UTAUT postquestionnaire corroborate with why perceptions of EE decreased after the assessment. There was some frustration for some participants with the touch screen sensitivity or *touch screen delay*. Some felt the instructions were *clear*, whereas others felt they were not. The report of unclear instructions did not apply to the overall BrainFx SCREEN instructions but to certain instructions for specific tasks.

FC is the degree to which an individual believes that an organizational and technical infrastructure exists to support the use of the system [28]. This variable had the largest effect on the BI. Before using the BrainFx SCREEN, some participants subjectively reported that they had reservations about the unknown, “anxiety about what it will be like,” and uncertainty about what to expect. It is reasonable that the participants felt supported by the CBA, organization, and other facilitators in the immediate environment during the assessment, which reduced their *fear of the unknown*. This could explain the statistically significant improvement in FC in the pre- and post-MGA.

**Figure 4:**
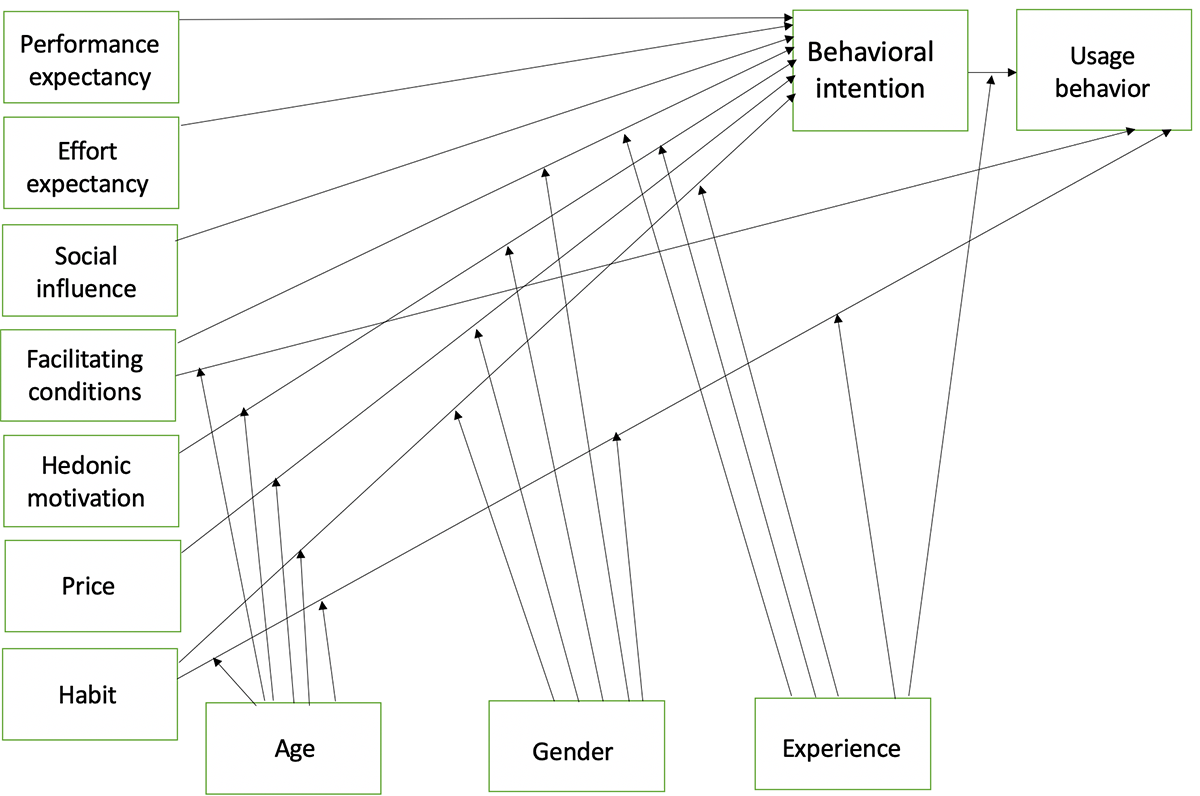
The Unified Theory of Acceptance and Use of Technology 2.

The thematic analysis also revealed some unexpected findings that could not be categorized into the variables of the UTAUT model. Some participants reported that the BrainFx SCREEN was *fun* and *engaging*. These experiences may fit better within the update to the UTAUT model, the UTAUT 2 (Figure 4) [48]. This model aims to provide a more consumer-based explanation of BI and use for technology by incorporating a number of additional latent variables, including price, habit, and hedonic motivation. Although the model is geared toward the consumer context, UTAUT 2 has been used in studies addressing technology in the health care context and is emerging in the technology acceptance literature [49].

As the BrainFx SCREEN does not cost the participants money, price would not be a factor that affects BI for this user group. As the screen is not intended to be used by the patient routinely, habit is also not an appropriate variable to be included in the research model. On the basis of the thematic analysis responses, hedonic motivation may be a variable that may influence BI in this study. Hedonic motivation is defined as “the fun or pleasure derived from using technology, and it has been shown to play an important role in determining technology acceptance and use” [50]. The perceived enjoyment of technological innovation has been found to influence technology acceptance and use directly for consumers [50]. Statements within the qualitative data analysis involving one’s enjoyment of the BrainFx SCREEN fit better within the definition of hedonic motivation than the other latent variable definitions, which suggests that this may have been an unaccounted factor that unexpectedly influenced BI. Hedonic motivation may be a variable that warrants further consideration when considering technology acceptance and usability in health care and potentially military contexts.

Another unexpected observation was that participants may not have understood the purpose of cognitive assessments in general. Even with written and verbal explanations of the purpose of and reason for the BrainFx SCREEN that was similar to or more comprehensive than that provided in a typical clinical environment, it was observed during data analysis that some participants did not fully understand these explanations. Some of the qualitative responses indicated that participants felt this tool was for the purpose of improving their cognition or a brain game. This may be due to the myriad of tablet-based apps currently on the market being advertised as mHealth tools, despite limited evidence of their efficacy for improving cognitive status [24]. It is also possible that some participants experienced cognitive impairment that hindered their ability to fully comprehend the instructions and explanations. Additional comorbidities, aside from mTBI and PTSD, that may adversely affect cognition and presented among the participants included other mental health diagnoses, chronic pain, fatigue, sleep challenges, and use of prescription medications. As stated, the presence of comorbid conditions among military personnel and veterans is not uncommon. Although the indicators for PE showed good reliability and validity, it is possible that a misunderstanding of the purpose of the BrainFx SCREEN could negatively affect this. This serves as a reminder that as researchers and health care professionals alike, the purpose of assessment and screening tools must be explained explicitly, especially with populations who may be experiencing cognitive impairment. 

Of note, one participant reported feeling disturbed by the images in the BrainFx SCREEN. Although the imagery within the assessment is generic and positive (eg, candy, animals, or plants), it is an important reminder that items within any assessment can potentially act as a trigger for a person experiencing PTSD and may increase levels of distress.

### Limitations of This Study

Although PLS-SEM is ideal for exploratory research and is flexible with its nonparametric lack of assumptions regarding data distribution, a number of limitations need to be considered. First, measurement errors always exist to some degree and are challenging to quantify accurately. The PLS-SEM bias refers to the tendency of the path model relationships to be frequently underestimated, whereas the parameters of the measurement model, such as the outer loadings, are overestimated when compared with covariance-based SEM. Measurement error can also be introduced by variables such as the participants’ understanding of the questionnaire items. As discussed, the level of understanding of the purpose of cognitive assessments may have been an issue, which raises questions about the participants’ understanding of other aspects. In addition, the administrative burden of the study when combined with other outcome measures attributed to the RCT with which this study was affiliated may have caused some participants to rush through final questionnaires or experience fatigue and a reduced level of engagement. Second, the lack of global goodness-of-fit measures is considered a drawback of PLS-SEM, which is unavoidable. Third, in the measurement model, BI had only one indicator variable. This made it impossible to evaluate it in a manner similar to the other latent variables. In the future, this could be resolved by adding additional items (indicators) to the UTAUT questionnaires related to BI. Finally, because the study was affected by a COVID-19–related shutdown, the original statistical power was not reached at 1% significance. The required sample size of a minimum of 24 participants was not attained, so the significance was 5% (N=21; R^2^=50%) [33]. Furthermore, the small sample size made it impossible to incorporate the moderator variables of age and gender, as was originally planned in the research model (Figure 2).

### Future Research

A range of future research endeavors would enhance the understanding of the relationship of the patient, whether military or civilians, with technological innovations. The technology acceptance and usability of the BrainFx SCREEN, as well as other assessments using digital health care technology, warrant evaluation within military and civilian health care and at multiple user levels, including patients, health care professionals, and organizations. This also extends to the use of virtual health care technologies where the patient is at a separate location from the health care professionals—a practice that is becoming increasingly widespread since the onset of the COVID-19 pandemic. It is important for health care professionals to become stakeholders in the process of adopting new health care technology. Studies with larger sample sizes may also allow for a research model with the ability to incorporate moderator variables, such as age, gender, voluntariness of use, and experience, as well as to investigate the effect of hedonic motivation as a latent variable.

The use of the UTAUT as a model for health care technology and patient user groups warrants continued investigation in both civilian and military settings. Furthermore, the appropriateness of the UTAUT and possibly other technology acceptance models within military contexts remain to be an area where research is scarce.

The limitation of the existing technology adoption models is the lack of task focus (ﬁt) between users, technology, and organization, which contributes to the mixed results in information technology evaluation studies [51]. Notably, within the military context, the environment and culture will have an effect on this at multiple user levels. The organization itself is considered a key factor in the effective use of information technology. To fully evaluate user acceptance of technology, the ﬁt between the user, the technology, and the organization needs to be evaluated together [52,53]. *Fit* needs to be integrated with existing technology models to better understand issues surrounding the implementation of new technology [53]. Multiple models and frameworks addressing technology acceptance and usability as well as fit exist, including the Task-Technology Fit model [54], Fit between Individuals, Task, and Technology framework [55], and Design-Reality Gap Model [56].

Information security has not been incorporated within technology adoption models or frameworks related to user acceptance. This may have important implications in both the military and clinical contexts. When users perceive that a particular technology provides features that prevent unauthorized access to the clinical-related database, they are more likely to trust and accept it [53]. The incorporation of information security and its involvement in technology acceptance and usability could be an interesting and relevant direction of research in military organizations.

### Conclusions

mTBI was labeled the *signature injury* of military conflicts during the War on Terror, in which National Atlantic Treaty Organization forces, including Canada, participated [3,57]. In addition, numerous military personnel and veterans from around the globe who have returned from deployments to this conflict continue to struggle with symptoms of PTSD either in isolation or comorbid with mTBI or TBI. Despite the plethora of research, publications, and attention that mTBI and PTSD have received in recent years, both in the military and sport contexts, many questions remain regarding the complexities of assessing and treating neurological symptomatology attributed to these diagnoses, including cognitive dysfunction. The BrainFx SCREEN appears to be a promising NCAT with good acceptability by CAF-SMs and veterans with crPTSD in this study. Future research is needed to address other factors of the BrainFx SCREEN, including its validity, reliability, effectiveness, feasibility, and sensitivity. As civilian and military health care systems increasingly integrate technological innovations to improve the services and care provided to their patients, research must continue to address the use of these novel assessments and interventions at the micro, meso, and macro levels.

## Abbreviations

AVE: average variance extracted
BI: behavioral intention
CAF-SM: Canadian Armed Forces service member
CBA: Certified BrainFx Administrator
CFHS: Canadian Forces Health Services
crPTSD: combat-related posttraumatic stress disorder
DSM-5: Diagnostic and Statistical Manual of Mental Disorders, 5th Edition
EE: effort expectancy
FC: facilitating conditions
HTMT: Heterotrait-Monotrait ratio
MGA: multigroup analysis
mHealth: mobile health
mTBI: mild traumatic brain injury
NCAT: computerized neurocognitive assessment tool
PCS: postconcussive symptom
PE: performance expectancy
PLS: partial least square
PTSD: posttraumatic stress disorder
RCT: randomized controlled trial
SEM: structural equation modeling
SI: social influence
TBI: traumatic brain injury
UTAUT: Unified Theory of Acceptance and Use of Technology
